# Poly(trimethylene carbonate-*co*-L-lactide) electrospun scaffolds for use as vascular grafts

**DOI:** 10.1590/1414-431X20198318

**Published:** 2019-08-12

**Authors:** D.I. Braghirolli, B. Caberlon, D. Gamba, JFTC. Petry, M.L. Dias, P. Pranke

**Affiliations:** 1 Laboratório de Hematologia e Células-tronco, Faculdade de Farmácia, Universidade Federal do Rio Grande do Sul, Porto Alegre, RS, Brasil; 2 Programa de Pós-Graduação em Fisiologia, Universidade Federal do Rio Grande do Sul, Porto Alegre, RS, Brasil; 3 Instituto de Química, Universidade Federal do Rio Grande do Sul, Porto Alegre, RS, Brasil; 4 Instituto de Macromoléculas Professora Eloisa Mano (IMA), Universidade Federal do Rio de Janeiro, Rio de Janeiro, RJ, Brasil; 5 Instituto de Pesquisa com Células-tronco, Porto Alegre, RS, Brasil

**Keywords:** Poly(trimethylene carbonate-*co*-L-lactide), Vascular scaffolds, Mesenchymal stem cells, Endothelial progenitor cells, Smooth muscle cells

## Abstract

Currently, there is great clinical need for suitable synthetic grafts that can be used in vascular diseases. Synthetic grafts have been successfully used in medium and large arteries, however, their use in small diameter vessels is limited and presents a high failure rate. In this context, the aim of this study was to develop tissue engineering scaffolds, using poly(trimethylene carbonate-*co*-L-lactide) (PTMCLLA), for application as small diameter vascular grafts. For this, copolymers with varying trimethylene carbonate/lactide ratios – 20/80, 30/70, and 40/60 – were submitted to electrospinning and the resulting scaffolds were evaluated in terms of their physicochemical and biological properties. The scaffolds produced with PTMCLLA 20/80, 30/70, and 40/60 showed smooth fibers with an average diameter of 771±273, 606±242, and 697±232 nm, respectively. When the degradation ratio was evaluated, the three scaffold groups had a similar molecular weight (*M*_w_) on the final day of analysis. PTMCLLA 30/70 and 40/60 scaffolds exhibited greater flexibility than the PTMCLLA 20/80. However, the PTMCLLA 40/60 scaffolds showed a large wrinkling and their biological properties were not evaluated. The PTMCLLA 30/70 scaffolds supported the adhesion and growth of mesenchymal stem cells (MSCs), endothelial progenitor cells, and smooth muscle cells (SMCs). In addition, they provided a spreading of MSCs and SMCs. Given the results, the electrospun scaffolds produced with PTMCLLA 30/70 copolymer can be considered promising candidates for future applications in vascular tissue engineering.

## Introduction

Cardiovascular disease (CVD) affects a large number of people worldwide, causing about 30% of all global deaths. The treatment of CVD involving vessel lesions can include surgical intervention, where a vascular graft is implanted (1). Synthetic vascular grafts have been widely used in bypass surgeries of large and medium caliber vessels. However, when vessels with an inner diameter of less than 6 mm are affected, these grafts present a high failure rate (2). Thrombosis, intimal hyperplasia and/or infection are the most common causes of failure of these vascular grafts (3). Therefore, vascular tissue engineering (VTE) can be considered an area of growing importance given the great need for vascular grafts, mainly for small vessels (1). In VTE, scaffolds populated with vascular cells are used as temporary grafts and favor the regeneration of the damaged tissue.

Scaffolds for use as vascular grafts should exhibit adequate mechanical properties and good interaction with the host tissue, in addition to supporting the development of endothelial and smooth muscle cells (4). Electrospun scaffolds produced from aliphatic polyester have been widely used in tissue engineering. However, polyesters such as poly(L- or DL-lactide) (PLA) and polyglycolide (PGA) are stiff materials and do not show an elasticity compatible with vascular applications (5). Moreover, the degradation of these polymers by bulk erosion causes a large loss of scaffold strength (6).

Poly(trimethylene carbonate) (PTMC) is a biodegradable elastomer that can be applied for the production of scaffolds in soft tissue engineering. This polymer shows high extensibility and shape recovery capability, besides degrading into nonacid and nontoxic residues (5,6). Various studies have demonstrated that the association between polyesters and PTMC allows for the creation of materials with mechanical characteristics and a degradation profile appropriate for vascular reconstruction (7,8). The physicochemical properties of the copolymer poly(trimethylene carbonate-*co*-L-lactide) (PTMCLLA) have been adjusted by varying the molar ratio of lactide (LA) and trimethylene carbonate (TMC) monomers (9) in order to obtain an adequate vascular scaffold.

The wall of blood vessels is organized in three concentric layers, each being formed by a different cellular type. The innermost layer, in contact with the blood, is the endothelial layer and is formed by endothelial cells. The middle layer is formed by smooth muscle cells and the outermost layer, called the adventitia layer, is formed by fibroblasts (10). To mimic the structure and functions of native vessels, it is important that scaffolds be compatible with the development of these three types of cells.

Based on the quoted data, in this work, scaffolds were produced with PTMCLLA (with different molar ratios of LA and TMC) by the electrospinning technique. The produced scaffolds were evaluated in terms of *in vitro* physicochemical and biological properties. The mechanical properties and degradation rate of the PTMCLLA scaffolds were studied. The biocompatibility of the PTMCLLA scaffolds was demonstrated through the (*in vitro*) interaction of endothelial progenitor cells, smooth muscle cells, and mesenchymal stem cells with their structure.

## Material and Methods

### Synthesis and characterization of TMC-LA polymers

L-lactide and 1,3-trimethylene carbonate in different TMC/LLA feed ratios (20/80, 30/70, or 40/60 mol %) were introduced in a Schlenk flask previously dried in an oven at 100°C for 24 h. The flask was charged with the desired amount of the octoate initiator to obtain a monomer/initiator molar ratio of 2,500 and then immersed in an oil bath at a selected temperature (180°C) under magnetic stirring. The copolymerization was carried out for 2 h. At the end of the reaction time, the product was fast-cooled to interrupt the reaction. The solid was then dissolved in chloroform and precipitated in cold ethanol, filtered, and dried in an oven at 50°C for 24 h (11).

The number-average (*M*_n_) and weight-average (*M*_w_) molecular weight of the copolymers were determined by means of gel permeation chromatography (GPC) in a Shimadzu LC 20 (Japan) equipped with a set of two Phenogel columns and a RID-20A differential index detector. Monodisperse polystyrene standards were used for calibration, and chloroform (CHCl3) was used as the solvent. Analyses were carried out at 30°C with a flow rate of 1.0 mL/min. The molecular weights were determined by Shimadzu software.

### Scaffolds production

Polymeric scaffolds were prepared by the electrospinning technique (12). Initially, different combinations of solvents, concentrations of polymer solutions, and electrospinning parameters were evaluated. The parameters were tested until homogeneous and smooth fibers were obtained. The parameters chosen to produce the scaffolds are presented in Table 1. The fibers were collected on 15 mm diameter cover slips and sterilized by UV radiation for 2 h.

**Table 1:** Parameters tested for scaffold production.

Copolymer	Polymeric solution parameters		Electrospinning parameters		
	Concentration	Solvent	Flow rate (mL/h)	Voltage (kV)	Distance between needle and plate collector (cm)
PTMCLLA 20/80	16%	DCM:DMF (7:3)	1.74	19	15
PTMCLLA 30/70	14%	DCM:DMF (7:3)	1.74	21	15
PTMCLLA 40/60	14%	HFIP	1.14	14	18

DCM: dichloromethane, DMF: dimethylformamide; HFIP: 1,1,1,3,3,3-hexafluoro-2-propanol.

### Scaffold characterization

#### *Morphology and fiber diameter*

The morphology of the electrospun fibers was analyzed by scanning electron microscopy (SEM) (Zeiss Evo 50, Germany) with an accelerating voltage of 10 kV after sputter coating with platinum. The average diameter was determined in SEM images by the measurement of 30 fibers from each sample (n=3) using ImageJ 1.46r software (NIH, USA).

#### In vitro *degradation*

The PTMCLLA scaffolds (12–20 mg) were immersed in 10 mL phosphate buffer saline (PBS) in individual falcon tubes and maintained at 37°C in an orbital shaker (100 rpm). The PBS solution was changed every 4 days. The scaffolds were removed from the PBS at regular time intervals and dried at 30° for 24 h. The changes in the polymeric molecular weight of the samples were estimated by a size exclusion chromatography module (Viscotek VE 2001) equipped with a refraction index detector (13). The samples were dissolved in tetrahydrofuran, filtered, and eluted at a flow rate of 1 mL/min at 45°C. A calibration curve made with polystyrene standard was used for molecular weight determination.

#### *Mechanical testing*

Tensile stress-strain measurements were evaluated in a DMA Q800 (*TA* Instruments, USA) equipped with a tension film clamp and using controlled force mode. Young's modulus, stress at break, and elongation at break were measured. The analyses were performed using rectangular strips (5×20×0.06 mm) cut from the scaffolds. The assays were carried out at a constant temperature (37°C) with ramp force of 0.5 N/min until 18 N maximum load, under 0.005 N static load (n=4) (14).

### Mesenchymal stem cell isolation and culture

Mesenchymal stem cells (MSCs) were isolated from deciduous teeth pulp and characterized in accordance with Werle and collaborators, after approval by the Ethics Committee of the Universidade Federal do Rio Grande do Sul (15). MSCs were cultivated in Dulbecco's Modified Eagle's Medium (DMEM) (Sigma-Aldrich, USA) containing 2.5 g/L Hepes (Sigma-Aldrich), supplemented with 10% fetal bovine serum (FBS) (ThermoFisher Scientific, USA) and 1% penicillin and streptomycin (ThermoFisher Scientific), at 37°C, in 5% CO2. The culture medium was changed every 3–4 days. When a confluence of 90% was reached, the cells were detached with 0.5% trypsin-EDTA solution (Sigma-Aldrich).

### Endothelial progenitor cells isolation and culture

Endothelial progenitor cells (EPCs) were isolated from umbilical cord blood after approval by the Ethics Committee of the Universidade Federal do Rio Grande do Sul and Ethics Committe of Moinhos de Vento Hospital. The EPCs were isolated and characterized, as previously described (16). The cells were cultivated in EGM-2 medium (Lonza) and supplemented with 15% FBS. When the cells reached 90% confluence, a passage using Tryple Express (ThermoFisher Scientific) reagent was carried out.

### Smooth muscle cell culture

Primary aortic smooth muscle cells (SMCs) were ceded by the Heart Institute (InCor - University of São Paulo Medical School - HCFMUSP). The cells were cultivated in DMEM supplemented with 20% FBS and 1% penicillin and streptomycin at 37°C, in 5% CO2. The culture medium was changed every 3–4 days. When 90% confluence was reached, the cells were detached with 0.5% trypsin-EDTA solution.

### Biological properties of scaffolds

The interaction between the PTMCLLA scaffolds and MSCs, EPCs, and SMCs was evaluated in terms of cellular adhesion, morphology, and viability during the 14 days of cultivation. For all analyses, the scaffolds were fixed in 24-well plates using a silicone o-ring and sterilized by UV irradiation for 2 h. Following this, trypsinized MSCs, EPCs, or SMCs were seeded onto the scaffolds at a density of 5×10^4^ cells/sample and incubated at 37°C, in 5% CO_2_ humidified atmosphere. Control cells were seeded and cultivated in wells without scaffolds.

#### *Cell adhesion*

Cell adhesion was evaluated by a colorimetric method using Cell Counting Kit-8 (CCK-8, Sigma-Aldrich) assay. After 3 h of cell seeding, the medium was removed and the samples were submitted to CCK-8 assay, in accordance with the manufacturer's instructions. The absorbance was read at 450 nm on a spectrophotometer (Multiscan FC, Thermo Scientific) and compared with a standard absorbance curve for each type of cell in a known concentration.

#### *Cell viability*

Cell viability was also evaluated by CCK-8 assay (16). After 1, 7, and 14 days of cultivation, the medium was removed and the cell viability was evaluated with CCK-8 kit, as described above. The cell growth rate (GR) was estimated by the following equation:

$$GR=\frac{OP14}{OP1}$$

where *OP14* and *OP1* are the optical density obtained by CCK-8 assay after 14 and 1 day of cell cultivation.

#### *Cell morphology*

After 24 h of cultivation, the samples were fixed with 4% paraformaldehyde for 30 min, washed with PBS, and incubated with 0.1% triton X-100, for 30 min, at room temperature. The cells were stained with 50 µg/mL rhodamine phalloidin (40 min) (Sigma-Aldrich) for actin and 0.5 μg/mL 4′,6-diamidino-2-phenylindole (DAPI) (1 min) for nuclei (14). The images were analyzed by confocal microscopy (Leica TCS SP5; ×63 lens, Germany).

### Statistical analysis

The results were analyzed and the data are reported as means±SD. The symmetry study of the distributions was performed using the Shapiro-Wilk test. The diameter of the fibers and the mechanical properties of the scaffolds were compared using Kruskal-Wallis followed by Dunn's post-test. The cellular adhesion and viability results were compared using ANOVA followed by Tukey’s post-test. Differences were considered significant when P<0.05.

## Results

### Synthesis of copolymers

The copolymers of TMC and LA were obtained in three different ratios: PTMCLLA 20/80, PTMCLLA 30/70, and PTMCLLA 40/60. The molecular weight of each copolymer is reported in Table 2.

**Table 2:** Molecular weight of poly(trimethylene carbonate-*co*-L-lactide) copolymers.

Ratio of TMC and LA in copolymers	Numeric molecular weight (*M*_n_)	Average molecular weight (*M*_w_)	*M*_w_/*M*_n_
20:80	69,700	132,000	1.89
30:70	56,500	92,000	1.63
40:60	22,800	36,800	1.61

TMC: trimethylene carbonate; LA: lactide.

#### *Physicochemical properties of scaffolds*

**Figure 1:**
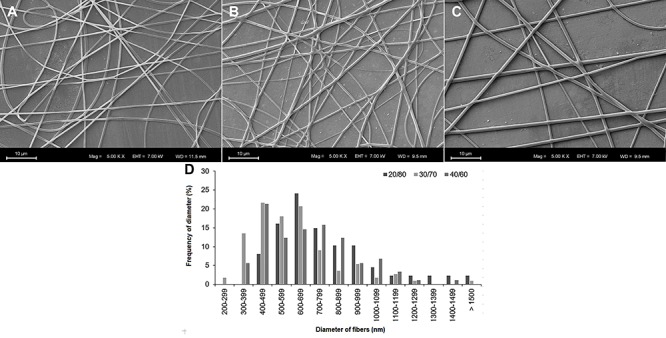
Scanning electron microscopy images of poly(trimethylene carbonate-*co*-L-lactide) (PTMCLLA) 20/80 (**A**), 30/70 (**B**), and 40/60 (**C**) (magnification ×5000, scale bars 10 µm). **D**, Distribution of diameter of fibers in the PLLATMC 20/80, 30/70, and 40/60 scaffolds.

Scaffolds were successfully produced by the electrospinning technique. Various solvents and concentrations of copolymers were tested to produce the fiber scaffolds, and the electrospinning parameters were varied. Using the parameters presented in Table 1, uniform fibers were obtained for the three types of copolymers. All the groups exhibited interconnected porous and smooth fibers, which were distributed randomly in the scaffolds structure (Figure 1A, B, and C). PTMCLLA 20/80 scaffolds showed fibers with an average diameter of 771±273 nm and 55% of the fibers had diameters between 500 and 799 nm (Figure 1D). Meanwhile, PTMCLLA 30/70 exhibited fibers with an average diameter of 606±242 nm, distributed mainly in the range of 300 to 799 nm (61%). The PTMCLLA 40/60 had fibers with an average diameter of 697±232 nm, with 76% of the fibers showing diameter between 400 and 899 nm. The PTMCLLA 30/70 fibers were significantly smaller than the other groups (P<0.05).

**Figure 2:**
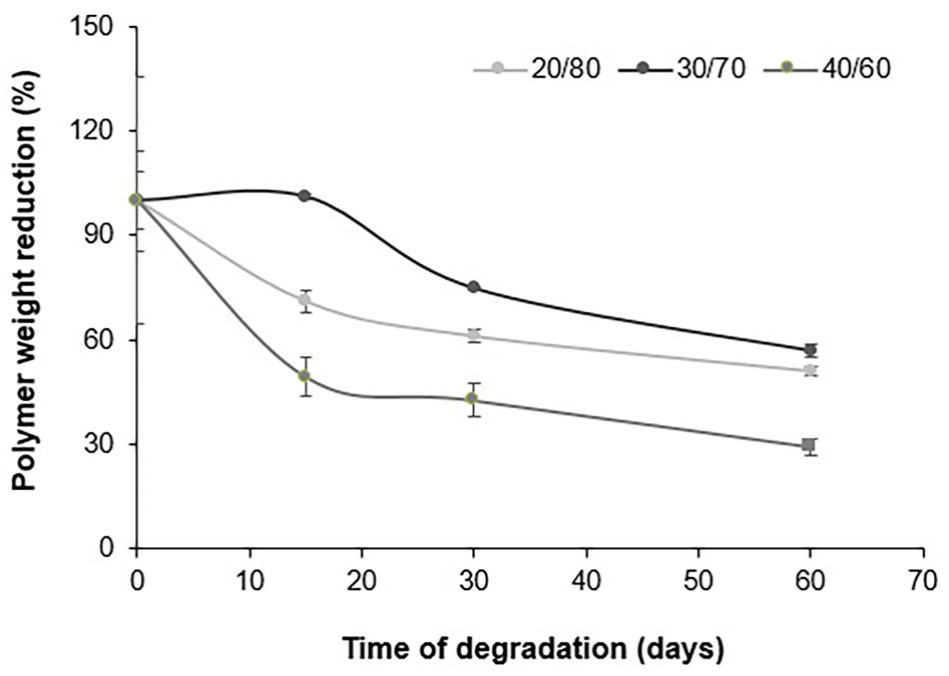
Average molecular weight (*M*_w_) of electrospun poly(trimethylene carbonate-*co*-L-lactide) (PTMCLLA) 20/80, 30/70, and 40/60 scaffolds, during the 60-day degradation assay. Data are reported as means±SD.

The degradation profile of the three scaffold groups was evaluated for 60 days, through the evaluation of the polymer molecular weight. On the 60th day of analysis, the molecular average weight (*M*_w_) of the three copolymer scaffolds was similar. However, the degradation profile was different between some groups (Figure 2). The copolymer PTMCLLA 20/80 and 30/70 scaffolds presented a similar reduction of *M*_w_ over the analyzed period. The PTMCLLA 20/80 and the PTMCLLA 30/70 scaffolds showed a 48 and 43% decrease in *M*_w_, respectively. Meanwhile, the scaffolds produced with PTMCLLA 40/60 presented a greater reduction of *M*_w_ in the first 7 days (about 42%) of the assay. After this time, this group showed a gradual reduction of molecular average weight, reducing by about 70% their initial *M*_w_.

**Figure 3:**
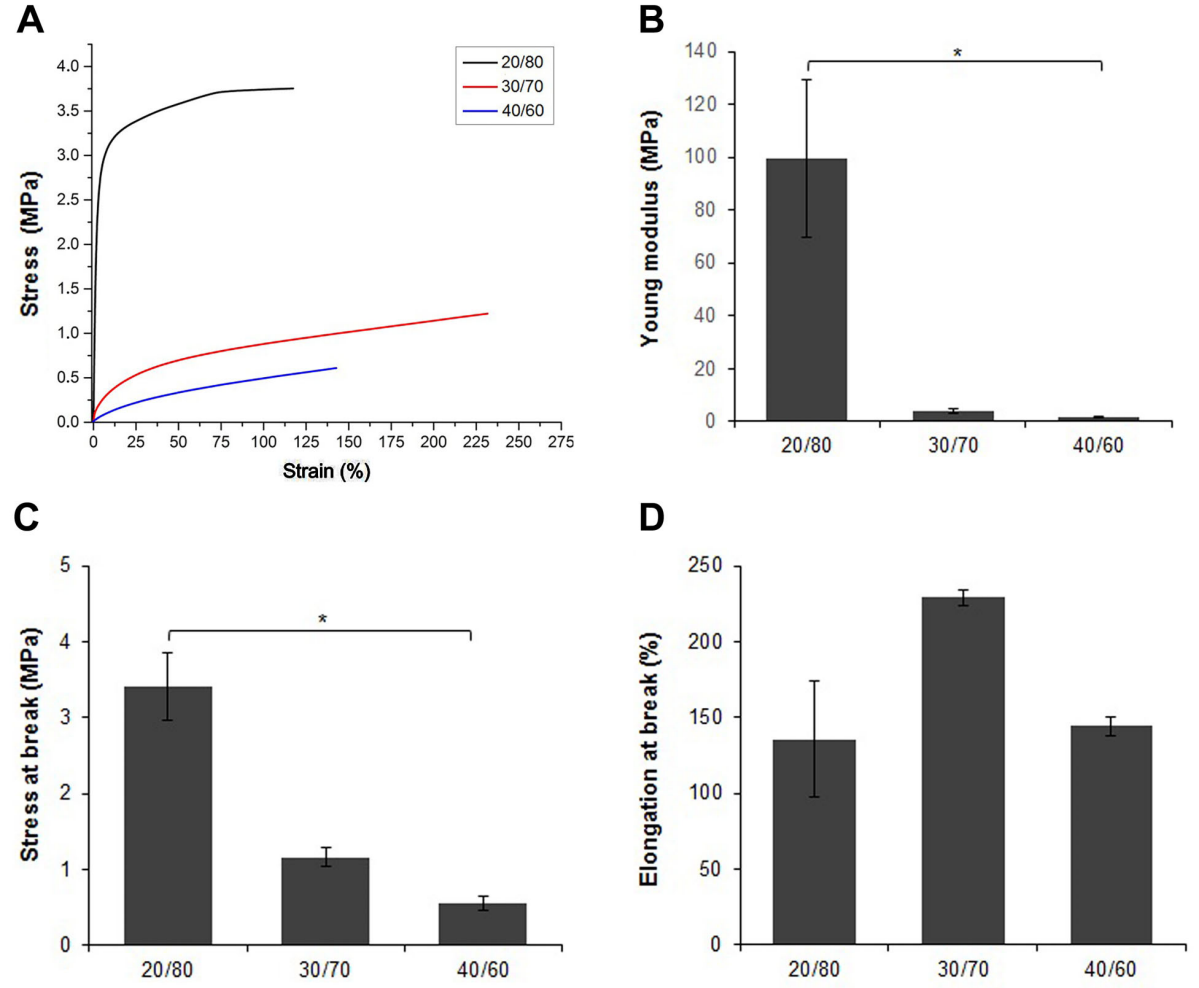
Mechanical properties of poly(trimethylene carbonate-*co*-L-lactide) (PTMCLLA) 20/80, 30/70, and 40/60 scaffolds. Stress-strain representative curve (**A**), Young modulus (**B**), stress at break (**C**), and elongation at break (**D**). Data are reported as means±SD. *P<0.05 between the groups (Kruskal-Wallis followed by Dunn's post-test).

Figure 3 presents stress-strain curves and derived tensile properties of the PTMCLLA electrospun scaffolds. The Young modulus and the stress at break varied in accordance with the ratio of the TMC and LA in copolymers. The Young modulus of the scaffolds reduced with the increase of TMC proportion in the copolymer: 99.5±30, 4.2±0.8, and 1.6±0.3 MPa for PTMCLLA 20/80, 30/70, and 40/60, respectively. This mechanical parameter was significantly different between the scaffolds produced with PTMCLLA 20/80 and PTMCLLA 40/60. Similar behavior was also observed for the stress at break. Meanwhile, the elongation at break did not appear to depend on the composition of the copolymers. There was no significant difference between the three scaffold groups and they all showed an elongation at break higher than 130%.

#### *Biological properties*

The MSCs, endothelial progenitor cells and SMCs were seeded onto the three scaffold groups and culture plate. The biological compatibility of the scaffolds was evaluated in terms of cell adhesion, morphology, and viability.

When the seeded PTMCLLA 40/60 scaffolds were incubated at 37°C, they showed severe wrinkling early within the first three h of cultivation. The area of this scaffold was greatly reduced, making it difficult to carry out the biological assays in this group. Thus, the PTMCLLA 40/60 scaffold was removed from the assays.

**Figure 4:**
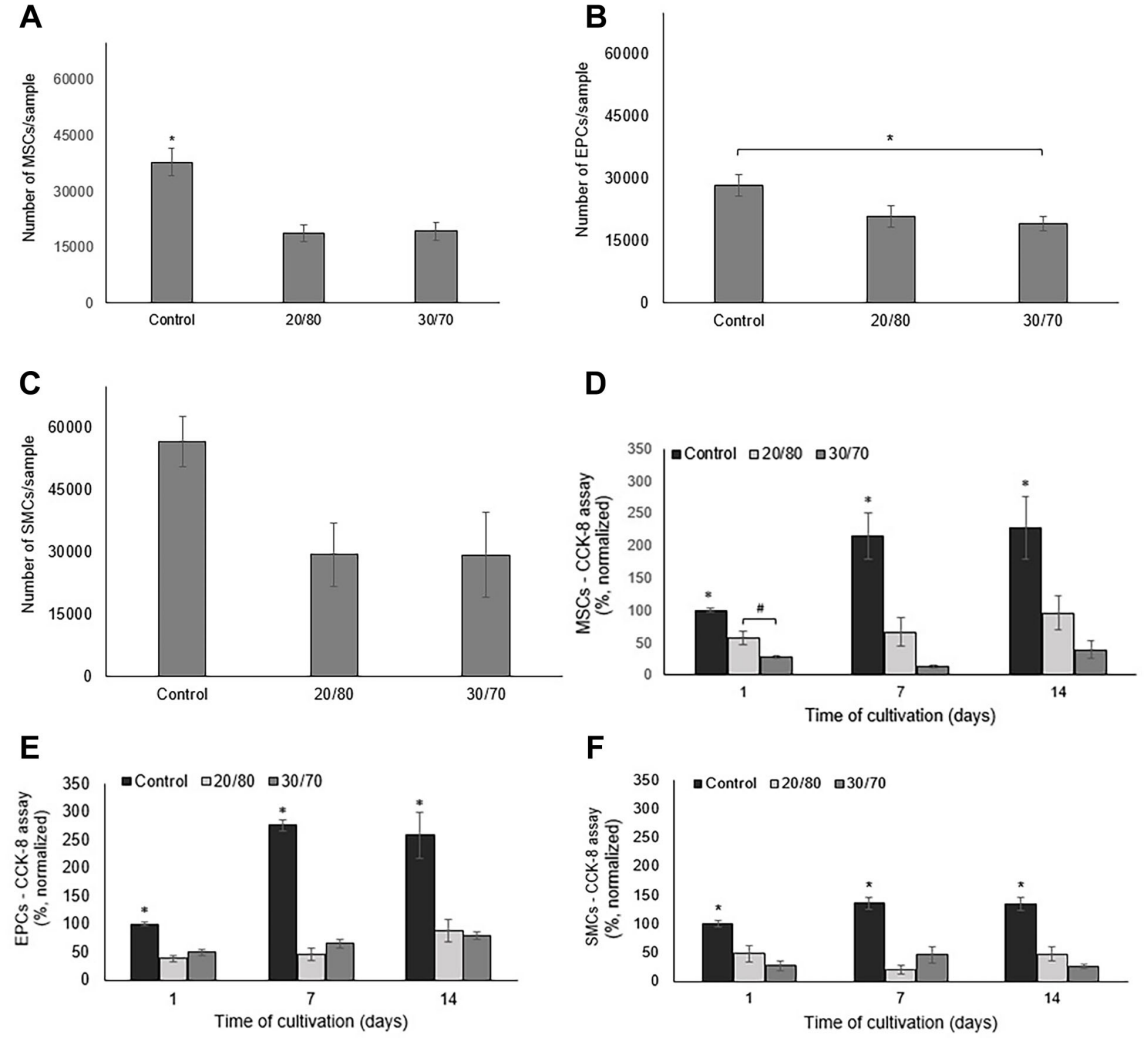
Adhesion of mesenchymal stem cells (MSCs) (**A**), endothelial progenitor cells (EPCs) (**B**), and smooth muscle cells (SMCs) (**C**) in the electrospun poly(trimethylene carbonate-*co*-L-lactide) (PTMCLLA) 20/80 and 30/70 scaffolds and in the culture plate (control group) after 3 h of incubation. Viability of MSCs (**D**), EPCs (**E**), and SMCs (**F**) during 14 days of cultivation in the scaffolds and in the culture plate. Data are reported as means±SD. *P<0.05 between the control and the other groups, ^#^P<0.01 between the PTMCLLA 20/80 and 30/70 groups (ANOVA followed by Tukey’s post-test).

The adhesion of cells to the scaffold surface was estimated by CCK-8 test, using a calibration curve. In all scaffold groups, a great number of adhered cells was observed in the control group (culture plate) (Figure 4A, C, and E). However, the different types of cells (MSCs, EPCs, and SMCs) also successfully adhered to the scaffold surface. All the types of cells showed a similar initial adhesion on the PTMCLLA 20/80 and PTMCLLA 30/70 scaffolds (P>0.05).

Cell viability was estimated during a period of 14 days by evaluating the mitochondrial activity by CCK-8. The increase rate of viable cells over 14 days was highest in the control group for all the cellular types (Figure 4B, D, and F) (P<0.05). Meanwhile, the different cell types presented different behavior in relation to the two scaffold groups. A greater number of viable MSCs was observed in the PTMCLLA 20/80 than the PTMCLLA 30/70 scaffold. However, the growth rate of MSCs between the first and last day of cultivation was similar between the two groups: 1.6 for PTMCLLA 20/80 and 1.4 for PTMCLLA 30/70. The control had a growth rate of 2.3.

The number of viable EPCs in both types of scaffolds was similar during the 14 days of cultivation (P>0.05). The growth rate of the EPCs between days 1 and 14 was 2.3 for PTMCLLA 20/80 and 1.6 for PTMCLLA 30/70. Meanwhile, the control group showed a growth rate of 2.5 for the EPCs.

A similar number of SMCs was observed for the PTMCLLA 20/80 compared to the PTMCLLA 30/70 scaffolds. This cellular type did not show an expressive increase in the number of cells on the scaffolds and on the culture plate. The growth rate, between days 1 and 14 for both scaffold groups was about 1.0 and 1.3 for the control group.

**Figure 5:**
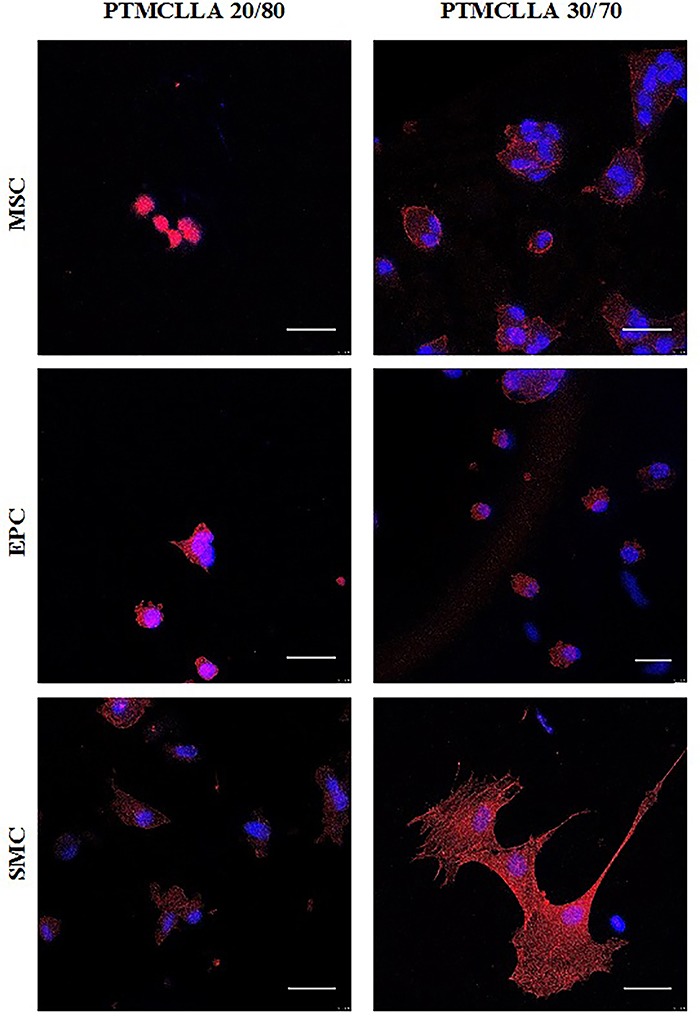
Confocal images of mesenchymal stem cells (MSCs), endothelial progenitor cells (EPCs), and smooth muscle cells (SMCs) cultivated on the poly(trimethylene carbonate-*co*-L-lactide) (PTMCLLA) 20/80 and 30/70 scaffolds for 24 h. The cells were stained with DAPI for nuclei (blue) and rhodamine-phalloidin for cytoskeleton (red). Original magnification ×63, scale bar 30 µm).

The morphology of adhered cells to the scaffold surface was analyzed by confocal microscopy after 24 h of cultivation (Figure 5). The MSCs exhibited a round morphology on the PTMCLLA 20/80 scaffolds. These cells had a slightly more developed cytoskeleton when cultivated on the PTMCLLA 30/70 scaffolds. The EPCs presented a similar morphology on the PTMCLLA 20/80 and 30/70 scaffolds. Cells showed a round cytoskeleton in both groups. Meanwhile, the SMCs exhibited a higher spreading rate on the scaffolds than the other cell types, mainly on the PTMCLLA 30/70 surface. The morphology of the SMCs indicates a better adaptation of these cells to biomaterials.

## Discussion

The great clinical need for vascular grafts and the lack of adequate substitutes for vessels of small diameters has driven research in the area of vascular tissue engineering. The aim of VTE is to create biocompatible vascular scaffolds that favor cell development and, when implanted, transform into autologous tissue, improving its functions (4). However, the development of a suitable vascular scaffold is a very challenging task. A vascular substitute should exhibit suitable physicochemical and biological characteristics in order to successfully fulfill all vessel functions after implantation. It must be biocompatible and able to support cellularization, both integrating and being indistinguishable from the host tissue. In addition to the biological properties, the scaffolds must be sufficiently elastic to withstand hemodynamic stress, without permanent changes in their structure (3).

Electrospinning has been successfully used in VTE. It facilitates the fabrication of flat sheets and hollow tubes with different diameters besides the fact that scaffolds mimic the vascular extracellular matrix. Scaffolds are formed by random fibers, with diameter range similar to natural collagen fibers, and they exhibit adequate porosity, which facilitates the development of cells in their structure (14,15). Synthetic polymers, mainly polyesters, are used for the development of scaffolds through electrospinning (12,17). However, for vascular tissue, polyesters such as PGA and PLA are rather stiff and fragile, requiring combination with more elastic materials (18). Trimethylene carbonate is a part of the shape-memory polymers class. This elastomer polymer has the intrinsic ability to recover its original shape after physical modifications (9). In addition, TMC copolymers show good hemocompatibility. Yang and coworkers showed that copolymers composed of TMC and polyesters such as L-lactide (LLA), DL-lactide (DLLA), or caprolactone (CL) present very low hemolytic ratios, indicating their good compatibility with human blood (19).

In this study, three copolymers with different ratios of TMC and LLA were produced. The electrospinning process was successfully carried out with each copolymer, also forming three scaffold groups. All the scaffolds exhibited interconnected pores in their structure. This feature ensures cellular migration, as well as the passage of nutrients and O_2_ through the scaffold structure, enabling tissue development (3). The three scaffold groups showed fibers with a smooth surface. However, the PTMCLLA 30/70 scaffolds showed smaller diameter fibers than the other groups. In their work, Bao and collaborators found that the lower molar ratio of TMC in the PTMCLLA copolymer resulted in a smaller diameter of the electrospun fibers (9). In the present study, the variation in fiber diameter was not dependent on the TMC molar rate in the copolymers. Unlike the paper of Bao and collaborators, in this study, each copolymer was processed under different parameters for the production of the scaffolds. The concentration of the polymer in solution, the molecular weight of the polymer, and the conductivity of the solution are some of the parameters that affect the diameter of electrospun fibers (20). The PTMCLLA 30/70 solution presented a lower concentration than the solution of PTMCLLA 20/80. Meanwhile, the different solvent systems used for solubilizing the PTMCLLLA 30/70 and PTMCLLA 40/60 copolymers could have altered the conductivity of their solutions and, consequently, their electrospinnability. These factors have possibly contributed to the differences in the fiber diameters between the PLLATMC 30/70 and the other scaffold groups. Despite the difference in fiber diameter, the morphology of the three groups of scaffolds was similar.

Different studies have shown that the TMC homopolymer exhibits a slow rate of *in vitro* degradation (21,22). In addition, the materials made with TMC show degradation by erosion, losing mass and dimensions proportionally to the surface area. Therefore, these materials are able to preserve their structural integrity and mechanical properties for a long time, supporting complete tissue formation (6). Yang and coworkers (22) showed that when the TMC is associated with polyesters, this characteristic is maintained. In their study, the mass of scaffolds made with PTMCLLA did not show great variation during the 20 weeks of the *in vitro* degradation assay. However, when GPC analysis was performed, changes in polymer molecular weight were observed. In the present study, the polymer average molecular weight also changed during the assay for all the scaffold groups, corroborating the data of Yang and collaborators. The scaffolds of PTMCLLA 20/80 and 30/70 showed a similar degradation profile and the reduction of polymer *M*_w_ was graded over the course of the assay. Meanwhile, the PTMCLLA 40/60 scaffolds exhibited a great rate of *M*_w_ reduction during the first 7 days, followed by a proportional degradation until 60 days. It is already well established that the ester bonds of lactyl units in PTMCLLA copolymers are more susceptible to hydrolytic cleavage than the carbonate bonds (5,22,23). Thus, it was expected that the PTMCLLA 40/60 scaffolds presented a higher resistance to degradation than the other groups, since they have a higher proportion of TMC in their composition. The observed behavior may have occurred because of the low molecular weight of this polymer and because of the presence of smaller polymeric chains.

The DMA analysis showed that the PTMCLLA 20/80 scaffolds had a stress-strain behavior typical of polyesters (14). The high modulus and stress at break exhibited by this group reflected their stiffness. As expected, the increase in the TMC content in the copolymers made the scaffolds more flexible. The increase of the TMC ratio reduced the Young modulus and the stress at break of the scaffolds. These results are consistent with other studies and demonstrate that the scaffolds with higher TMC content show a behavior similar to rubbery materials (9,24). Despite the reduction of strength, the PTMCLLA 30/70 and 40/60 scaffolds presented stress at break closer to the value found in the literature for native vessels (1.55±0.4 MPa) (8).

Biological characterization was conducted with all the scaffold groups. However, during the cultivation period, the PTMCLLA 40/60 presented a large shrinkage in size and was excluded from these assays. Ji and coworkers also observed this behavior for PTMCLLA electrospun scaffolds. Those authors were only able to evaluate the biocompatibility of scaffolds produced with PTMCLLA 15/85 (25).

Cellular adhesion is the first step of cell-scaffold interaction. This step is crucial for the success of biological scaffolds (26). Scaffolds should favor the adhesion of the cells so that they can proliferate and secrete extracellular matrix, organizing the tissue. In a similar way, the scaffolds made with PTMCLLA 20/80 and 30/70 copolymers supported the adhesion of MSC, EPC, and SMC. The number of adhered cells in the scaffolds was lower than in the culture dish for the three cellular lines. These results were expected as the commercial culture plates represent a gold standard surface for cell culture. Stefani and Cooper-White also observed this finding for MSCs and PCL/PTMCLLA electrospun scaffolds. The authors reported that in relation to the culture plates, only 3/4 of the MSCs seeded on the PCL/PTMCLLA scaffolds remained adherent to their surface (8).

Once adhered to the scaffolds, MSCs and EPCs showed an increase in absorbance of the CCK-8 assay, suggesting that proliferation of these cells occurred. The PTMCLLA 20/80 exhibited a large number of viable MSCs on the first day. This group also showed a larger number of viable MSCs than the PTMCLLA 30/70 on days 7 and 14. However, the differences were not significant. When EPCs were evaluated, no statistical differences were observed between the scaffold groups. These results suggest that the variation of TMC from 20 to 30% in the PTMCLLA composition did not have an impact on the proliferation of cells and that both electrospun scaffold types can support the growth of MSCs and EPCs. These results are similar to those of Dargaville and co-workers. Those authors evaluated the development of MSCs on electrospun scaffolds produced with copolymers containing from 30 to 70% TMC and no difference was found among the groups (5). The difference of fiber diameter between the two groups also caused no variation in MSCs and EPCs development. Meanwhile, SMCs did not exhibit a significant proliferation on the two types of scaffolds. The SMCs are already differentiated cells, which have a lower proliferation rate than immature cells like MSCs and EPCs. This characteristic is likely to have contributed to the observed result.

The good adaptation of the cell to scaffolds is crucial for tissue organization. When the scaffolds favor the adaptation of cells, they are able to establish several points of adhesion of their cytoskeleton with the fibers, resulting in a larger and more scattered cytoplasm. Therefore, onto these substrates, EPCs exhibit a cone shape and MSCs and SMCs have an elongated morphology (25,26). Cytoskeleton staining showed that the EPCs had similar behavior in both the scaffold groups. However, MSCs, and especially SMCs, had a more spreading morphology when cultivated on the PTMCLLA 30/70 scaffolds. Although the PTMCLLA 20/80 and 30/70 groups were similar in terms of cell proliferation, the difference in TMC content affected the adaptation of the MSCs and SMCs on the scaffolds. Ji and collaborators also found differences in the adaptation of fibroblasts cultivated on the PLLA and PTMCLLA 15/85 electrospun scaffolds. After 24 h, the fibroblasts cultivated on the scaffolds containing TMC exhibited a larger size than the fibroblasts cultivated on the PLLA scaffolds (25). This difference found in the present study is probably associated with scaffold flexibility. A substrate that is more flexible seems to be more favorable for adaptation of MSCs and SMCs than the stiff surfaces. The diameter of the nanofibers in the PTMCLLA 30/70 scaffolds may also have contributed to better adaptation of MSCs and SMCs. Fibers with smaller diameters offer a higher surface area, which can support a more homogenous cell distribution and spreading (27). However, some studies have demonstrated that small variations in diameter of fibers, such as those observed in the present study, do not modify the interaction of cells with the scaffolds (9,28). Therefore, it is believed that the differences in cell morphology obtained for the two groups of scaffolds have been caused mainly by their different rates of TMC and LA.

The PTMCLLA 30/70 copolymer allowed for the fabrication of flexible and resistant scaffolds, which favored the adaptation of vascular and stem cells. Although *in vivo* analysis is required, this study demonstrated that the electrospun PTMCLLA 30/70 scaffolds are potential biomaterials for use as vessel substitutes in surgical treatment of cardiovascular disease.

## Conclusions

In this study, scaffolds produced with different copolymers were evaluated for application as vascular grafts. PTMCLLA 30/70 and 40/60 scaffolds showed higher flexibility, showing greater appropriateness for application as vascular scaffolds. However, the 40/60 scaffolds presented a high shrinkage rate. The PTMCLLA 20/80 and 30/70 scaffolds supported the adhesion and growth of MSCs, EPCs, and SMCs. However, the MSCs and SMCs showed a greater spread morphology when cultivated on the PTMCLLA 30/70 scaffolds. Therefore, scaffolds produced with PTMCLLA 30/70 are promising candidates for future application in vascular tissue engineering.
